# Major adverse kidney events among chronic kidney disease patients with vitamin D deficiency

**DOI:** 10.3389/fnut.2025.1650514

**Published:** 2025-10-07

**Authors:** Yu-Min Lin, Chia-Li Kao, Kuo-Chuan Hung, Ting-Hui Liu, Tsung Yu, Mei-Yuan Liu, Jheng-Yan Wu, Chi-Lun Tsai

**Affiliations:** ^1^Division of Cardiology, Department of Internal Medicine, Chi Mei Medical Centre, Chiali, Tainan, Taiwan; ^2^Department of Anesthesiology, E-Da Hospital, I-Shou University, Kaohsiung City, Taiwan; ^3^Department of Anesthesiology, Chi Mei Medical Center, Tainan, Taiwan; ^4^Department of Psychiatry, Chi Mei Medical Center, Tainan, Taiwan; ^5^Department of Public Health, College of Medicine, National Cheng Kung University, Tainan, Taiwan; ^6^Department of Nutrition, Chi Mei Medical Center, Tainan, Taiwan; ^7^Department of Intensive Care Medicine, Chi Mei Medical Center, Tainan, Taiwan

**Keywords:** vitamin D deficiency, chronic kidney disease, major adverse kidney events, mortality, hospitalization

## Abstract

**Objective:**

This study aimed to evaluate the association of vitamin D deficiency (VDD) and major adverse kidney events (MAKEs) among patients with chronic kidney disease (CKD).

**Methods:**

We conducted a retrospective cohort study using the TriNetX Global Collaborative Network. Eligible participants were adults with CKD who had a vitamin D testing between January 01, 2010 and January 31, 2025. According to the status of vitamin level, individuals were classified into two groups, VDD group and control group. Propensity score matching (PSM) was applied to balance baseline characteristics between groups. The primary outcome was the risk of MAKEs during one-year follow-up, while secondary outcomes included all-cause mortality and all-cause hospitalization.

**Results:**

After PSM, 29,654 patients were included in each group. The VDD group was associated with a higher risk of MAKEs (hazard ratio [HR], 2.24; 95% confidence interval [CI], 2.08–2.41; *p* < 0.001). Stratified analyses revealed consistent relationship across multiple subgroups. Additionally, the VDD group was also associated with higher risks of all-cause mortality (HR, 1.92; 95% CI, 1.82–2.02; *p* < 0.001), and all-cause hospitalization (1.19; 95% CI, 1.14–1.25; *p* < 0.001).

**Conclusion:**

VDD in patients with CKD is associated with a significantly higher risk of MAKEs. The finding suggests that VDD may contribute to worse adverse kidney events and highlight the importance of vitamin D status in the clinical management.

## Introduction

Chronic kidney disease (CKD) is a growing public health concern and is strongly associated with premature cardiovascular disease (1, 2). CKD affects about 10–15% of adults worldwide and may progress to end-stage kidney disease (ESKD), requiring dialysis or transplantation. As kidney function progressively deteriorates, patients develop multiple complications, with one of the most significant being disturbances in mineral and bone metabolism, notably involving vitamin D (3).

Vitamin D is a fat-soluble prohormone crucial for calcium-phosphate balance, but it also exerts numerous extra-skeletal effects on immune regulation and cardiovascular health. In the kidney, the activation of vitamin D to its hormonal form (calcitriol) occurs, meaning renal impairment can directly perturb vitamin D metabolism (4). Vitamin D deficiency (VDD) is highly prevalent in CKD patients. In fact, over 80% of individuals with CKD have insufficient 25-hydroxyvitamin D levels (5, 6). This high prevalence is partly because even early-stage CKD (e.g., estimated glomerular filtration rate [eGFR] > 60 mL/min/1.73m^2^) is associated with declining vitamin D levels (6).

Patients with CKD often experience reduced sun exposure, dietary limitations, and increased urinary loss of vitamin D metabolites. Furthermore, early reductions in megalin expression impair the reabsorption of 25(OH)D from the glomerular filtrate, leading to decreased circulating levels (1). Consequently, CKD patients commonly develop secondary hyperparathyroidism and other sequelae of VDD. Beyond classical bone and mineral effects, vitamin D plays important roles in modulating the renin-angiotensin-aldosterone system (RAAS) and inflammation; VDD in CKD has been linked to faster disease progression and cardiovascular complications (7, 8).

Major Adverse Kidney Events (MAKEs) refer to a composite of critical renal outcomes, typically including sustained worsening of kidney function, initiation of renal replacement therapy (dialysis or transplantation), or kidney-related mortality (9). MAKEs are considered critical endpoints because they encompass the most serious outcomes for CKD patients and align with patient-centered clinical goals (9). While VDD has been associated with adverse outcomes in CKD (10, 11), its relationship with composite endpoints like MAKEs remains under-explored. Thus, the study aimed to examine the association between VDD and risk of MAKEs in patients with CKD.

## Methods

### Study design and database

The retrospective cohort study used data from the TriNetX Global Collaborative Network, a large-scale health research database that contains electronic medical records (EMRs) information from over 160 million patients across 140 healthcare organizations (HCOs) globally (12). The database encompasses a wide range of patient information, including demographics, medical diagnoses, clinical procedures, prescribed medications, lab test results, genetic information, and healthcare facility visit types. The TriNetX system provides researchers with immediate access to anonymized, consolidated health data from a broad spectrum of patients representing various geographic regions and ethnic backgrounds, collected from different healthcare settings including hospitals, primary care clinics, and specialized medical centers. The platform has received approval from the Western Institutional Review Board through a waiver, as it only processes aggregate statistical data rather than individual patient records. The researchers conducted this study following the Strengthening the Reporting of Observational Studies in Epidemiology (STROBE) guidelines (13).

### Study population and definition of eligible patients

We focused on adults diagnosed with CKD who had their vitamin D levels measured within a three-month window before their CKD diagnosis, spanning from January 01, 2010 to January 31, 2025. The index date was defined the vitamin D test date. Patients met the following criteria were enrolled in the study, including at least 18 years old and had a CKD diagnosis. In this study, CKD was defined using the ICD-10-CM code N18. Patients were stratified into two groups based on their vitamin D levels. The VDD group had vitamin D levels below 20 ng/mL, while the control group had vitamin D levels above 30 ng/mL (14–17). Individuals with vitamin D levels between 21–29 ng/mL, typically categorized as vitamin D insufficiency, were excluded from this study to ensure a clear comparison between deficient and sufficient vitamin D status. For robust data collection, each patient needed at least two EMR entries during the study period. To minimize protopathic and ascertainment bias, we excluded the primary outcome occurred prior to the index date (18) (Supplementary Table S1).

### Covariates

We selected covariates based on the clinical relevance, particularly those known to influence mortality and renal outcomes (19–22). We assessed baseline health status in accordance with contemporary medical understanding. For both groups, we extracted data on baseline characteristics and covariates from the year before the index date, including demographic factors (age, sex, race), clinical parameters (eGFR, albumin, HbA1c), comorbidities, and medications. The comorbidities included cardiometabolic conditions (hypertension, hyperlipidemia), nutritional status (malnutrition, obesity), metabolic disorders (type 2 diabetes mellitus), substance use patterns (nicotine dependence, alcohol-related disorders), respiratory conditions (chronic lower respiratory diseases), hepatic function (liver diseases), cardiovascular conditions (cerebrovascular diseases, atrial fibrillation and flutter, ischemic heart disease), autoimmune disorders (systemic lupus erythematosus), and malignancies (neoplasms). The medication profile analysis encompassed angiotensin-converting enzyme inhibitor (ACEi), angiotensin receptor blocker (ARB), beta-blocker, calcium channel blocker (CCB), diuretics, sodium-glucose cotransporter-2 inhibitor (SGLT2i), glucagon-like peptide-1 receptor agonist (GLP1RA), HMG CoA reductase inhibitors, erythropoietin, and finererone (Supplementary Table S2).

### Outcomes

Primary outcome in the study was MAKEs. Secondary outcomes included all-cause mortality, and all-cause hospitalization. MAKEs were characterized by ESKD, urgent dialysis initiation, or dialysis dependence (23, 24). Patient follow-up commenced the day after the index date and continued until their final clinical visit, death, or one-year post-index date, whichever occurred first (Supplementary Table S3).

### Statistical analysis

For baseline characteristics, continuous variables were presented as means with standard deviations (SDs), while categorical variables were expressed as frequencies and percentages. To minimize confounding bias and balance covariates between groups, we implemented propensity score matching (PSM) using a greedy nearest-neighbor algorithm, with a caliper width set at 0.1 pooled SD of the logit of the propensity score, consistent with the default TriNetX implementation. Because our study is based on a large real-world cohort derived from the TriNetX platform, direct statistical hypothesis testing (e.g., t-test, Mann–Whitney U test, or chi-square test) is not natively supported within the platform. Instead, we followed the current best practice for TriNetX-based studies by reporting continuous variables as mean ± SD and categorical variables as counts with percentages, and by evaluating covariate balance using standardized mean differences (SMDs), with an SMD < 0.1 indicating adequate balance (25). This approach is recommended for large-scale observational studies because *p* values are overly sensitive to very large sample sizes and may indicate statistical significance even for clinically negligible differences (25, 26). Using SMDs provides a more robust and interpretable measure of baseline comparability, consistent with the methodology adopted in recent studies published in JAMA Network Open and other high-impact journals. For variables with SMDs slightly exceeding 0.1, we further examined the absolute differences in clinical values to assess whether the imbalance was likely to be clinically meaningful. After matching, we conducted survival analysis using Kaplan–Meier curves and compared groups with log-rank tests (27, 28). The Cox regression models were used to determine hazard ratios (HRs) linking vitamin D status to outcomes (29). Additionally, we calculated E-values to assess how unmeasured factors might affect the findings (30). Statistical significance was defined as a two-sided *p* value below 0.05.

### Stratified analysis

We performed stratified analyses to examine the robustness of the primary outcome associations across varied subgroups. These analyses included age groups (18–64 vs. ≥ 65 years), sex differences (female vs. male), and CKD stage (stage 1–2 vs. stage 3–5), and nutritional status (albumin < 3.5 vs. ≥ 3.5 g/dL).

## Results

### Study flow diagram

**Figure 1:**
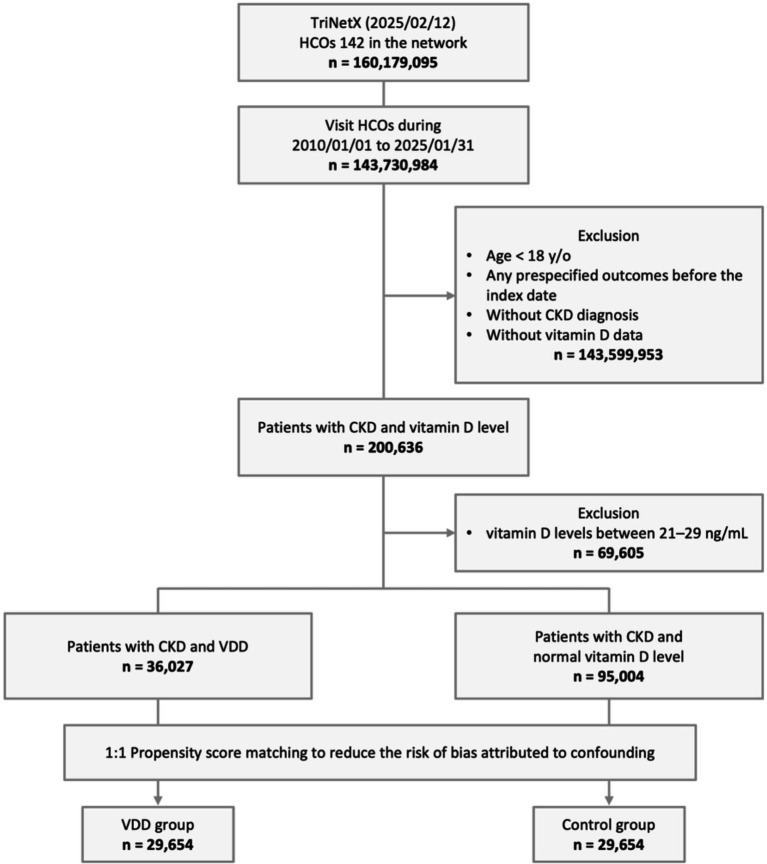
Study design and selection flow. HCO, healthcare organizations; CKD, chronic kidney disease; y/o, years old; VDD, vitamin D deficiency.

From a total population of 160,179,095 patients across 142 HCOs in the TriNetX network, we identified 143,730,984 individuals with visits between January 01, 2010, and January 31, 2025. We excluded 143,599,953 patients who met one or more of the following criteria: age below 18 years, occurrence of prespecified outcome before the index date, lack of vitamin D level measurements before the index date, or absence of CKD diagnosis. Of the remaining 200,636 eligible patients with both CKD and vitamin D measurements, 69,605 individuals with vitamin D levels between 21–29 ng/mL (vitamin D insufficiency) were excluded to ensure a clear contrast between deficiency and sufficiency groups. Among the remaining patients, 36,027 were categorized into the VDD group, while 95,004 comprised the control group with normal vitamin D levels. Following PSM, the final analysis included 29,654 patients in each group (Figure 1).

### Study population characteristics

Before PSM, there were significant differences between the VDD group (*n* = 36,027) and the control group (*n* = 95,004) (Table 1). Participants in the VDD group were younger (64.2 ± 16.2 vs. 72.5 ± 13.0 years) and had a lower proportion of female (44.9% vs. 54.3%). They also had higher prevalences of overweight and obesity (25.7% vs. 18.6%), malnutrition (10.0% vs. 5.0%), type 2 diabetes mellitus (49.6% vs. 35.9%), nicotine dependence (13.2% vs. 5.2%), alcohol related disorders (5.0% vs. 2.0%), liver disease (11.8% vs. 6.9%), and ischemic heart diseases (32.0% vs. 26.8%). Additionally, the VDD group had lower albumin levels (3.4 ± 0.8 vs. 3.9 ± 0.6 g/dL), eGFR (42.2 ± 24.3 vs. 45.4 ± 18.2 mL/min/1.73m^2^), and higher HbA1c levels (7.2 ± 2.3 vs. 6.5 ± 1.5%). Use of ACEis (26.1% vs. 20.1%), beta-blockers (50.1% vs. 39.7%), CCB (38.9% vs. 30.7%), and diuretics (50.7% vs. 38.3%) was also more frequent in the VDD group (Table 1).

**Table 1:** Baseline characteristics of included subjects.

Variables	Before matching			After matching		
	VDD group (*n* = 36,027)	Control group (*n* = 95,004)	Std diff	VDD group (*n* = 29,654)	Control group (*n* = 29,654)	Std diff
Age at index, years						
Mean ± SD	64.2 ± 16.2	72.5 ± 13.0	0.567	66.7 ± 15.2	66.8 ± 15.7	0.007
Sex, *n* (%)						
Female	16,166 (44.9)	51,597 (54.3)	0.190	13,894 (46.9)	13,828 (46.6)	0.004
Male	19,144 (53.1)	40,644 (42.8)	0.208	15,080 (50.9)	15,115 (51.0)	0.002
Race, *n* (%)						
White	16,156 (44.8)	61,134 (64.3)	0.399	14,757 (49.8)	14,757 (49.8)	< 0.001
Black or African American	10,990 (30.5)	14,149 (14.9)	0.379	7,765 (26.2)	7,877 (26.6)	0.009
Asian	1,567 (4.4)	7,718 (8.1)	0.157	1,474 (5.0)	1,372 (4.6)	0.016
Other race	1,077 (3.0)	2,110 (2.2)	0.048	830 (2.8)	854 (2.9)	0.005
Unknown race	5,489 (15.2)	8,772 (9.2)	0.184	4,319 (14.6)	4,296 (14.5)	0.002
Estimated glomerular filtration rate, mL/min/1.73m^2^						
Mean ± SD	42.2 ± 24.3	45.4 ± 18.2	0.152	42.4 ± 23.3	44.1 ± 20.5	0.077
≤ 45, *n* (%)	22,837 (63.4)	48,844 (51.4)	0.244	17,890 (60.3)	17,829 (60.1)	0.004
Albumin, g/dL						
Mean ± SD	3.4 ± 0.8	3.9 ± 0.6	0.702	3.5 ± 0.7	3.7 ± 0.6	0.316
≤3.5, *n* (%)	17,107 (47.5)	22,531 (23.7)	0.512	12,047 (40.6)	11,899 (40.1)	0.010
HbA1c, %1						
Mean ± SD	7.2 ± 2.3	6.5 ± 1.5	0.393	6.9 ± 2.0	6.7 ± 1.9	0.119
≥9, n (%)	4,249 (11.8)	3,273 (3.4)	0.319	2,295 (7.7)	2,279 (7.7)	0.002
Comorbidities, *n* (%)						
Hypertension	22,599 (62.7)	65,033 (68.5)	0.121	18,735 (63.2)	18,502 (62.4)	0.016
Hyperlipidemia	18,418 (51.1)	58,595 (61.7)	0.214	15,671 (52.8)	15,396 (51.9)	0.019
Overweight and obesity	9,252 (25.7)	17,703 (18.6)	0.170	6,953 (23.4)	6,877 (23.2)	0.006
Malnutrition	3,607 (10.0)	4,756 (5.0)	0.191	2,542 (8.6)	2,586 (8.7)	0.005
Type 2 diabetes mellitus	17,855 (49.6)	34,078 (35.9)	0.279	13,623 (45.9)	13,523 (45.6)	0.007
Nicotine dependence	4,766 (13.2)	4,958 (5.2)	0.280	2,936 (9.9)	2,960 (10.0)	0.003
Alcohol related disorders	1,810 (5.0)	1,895 (2.0)	0.165	1,135 (3.8)	1,091 (3.7)	0.008
Chronic lower respiratory diseases	7,593 (21.1)	17,754 (18.7)	0.060	6,153 (20.7)	6,009 (20.3)	0.012
Diseases of liver	4,244 (11.8)	6,545 (6.9)	0.169	3,050 (10.3)	2,948 (9.9)	0.011
Cerebrovascular diseases	5,276 (14.6)	10,977 (11.6)	0.092	4,158 (14.0)	4,124 (13.9)	0.003
Atrial fibrillation and flutter	6,581 (18.3)	17,069 (18.0)	0.008	5,583 (18.8)	5,694 (19.2)	0.010
Ischemic heart diseases	11,532 (32.0)	25,480 (26.8)	0.114	9,192 (31.0)	9,185 (31.0)	0.001
Systemic lupus erythematosus	476 (1.3)	1,191 (1.3)	0.006	377 (1.3)	373 (1.3)	0.001
Neoplasms	7,727 (21.4)	25,207 (26.5)	0.119	6,791 (22.9)	6,644 (22.4)	0.012
Medications, *n* (%)						
ACEis	9,406 (26.1)	19,064 (20.1)	0.144	7,091 (23.9)	6,965 (23.5)	0.010
ARBs	6,779 (18.8)	20,997 (22.1)	0.081	5,697 (19.2)	5,512 (18.6)	0.016
Beta blockers	18,049 (50.1)	37,746 (39.7)	0.210	13,940 (47.0)	13,910 (46.9)	0.002
Calcium channel blockers	14,014 (38.9)	29,169 (30.7)	0.173	10,728 (36.2)	10,557 (35.6)	0.012
Diuretics	18,274 (50.7)	36,433 (38.3)	0.251	14,040 (47.3)	13,723 (46.3)	0.021
SGLT2i	1,524 (4.2)	4,504 (4.7)	0.025	1,300 (4.4)	1,261 (4.3)	0.006
GLP1RA	1,133 (3.1)	3,528 (3.7)	0.031	984 (3.3)	977 (3.3)	0.001
HMG CoA reductase inhibitors	16,267 (45.2)	42,354 (44.6)	0.011	13,272 (44.8)	13,023 (43.9)	0.017
Erythropoietin	735 (2.0)	1,101 (1.2)	0.070	526 (1.8)	531 (1.8)	0.001
Finerenone	30 (0.1)	128 (0.1)	0.016	28 (0.1)	32 (0.1)	0.004

ACEi, angiotensin-converting enzyme inhibitor; ARB, angiotensin receptor blocker; ARNI, angiotensin receptor-neprilysin inhibitor; GLP1RA, glucagon-like peptide-1 receptor agonist; SGLT2i, sodium-glucose cotransporter-2 inhibitor; Std Diff, standardized difference; VDD, vitamin D deficiency. Standardized difference (Std diff) < 0.1 is considered a small difference.

After PSM, both the VDD (*n* = 29,654) and control (*n* = 29,654) groups were well balanced in baseline characteristics, as shown by standardized differences <0.1 for most variables. Their mean ages were comparable (66.7 ± 15.2 vs. 66.8 ± 15.7 years), and the proportions of females were nearly identical (46.9% vs. 46.6%). Comorbidities such as type 2 diabetes mellitus, hypertension, overweight and obesity, and chronic lower respiratory diseases were also similar between the two groups. Although the standardized differences for albumin and HbA1c slightly exceeded the 0.1 threshold, their actual values were closely aligned between groups (albumin: 3.5 vs. 3.7 g/dL; HbA1c: 6.9% vs. 6.7%), suggesting limited clinical relevance. Moreover, key categorical thresholds, such as albumin levels below 3.5 g/dL, were well balanced. This further supports the comparability of clinical characteristics between groups (Table 1).

### Primary outcome and stratified analysis

**Figure 2:**
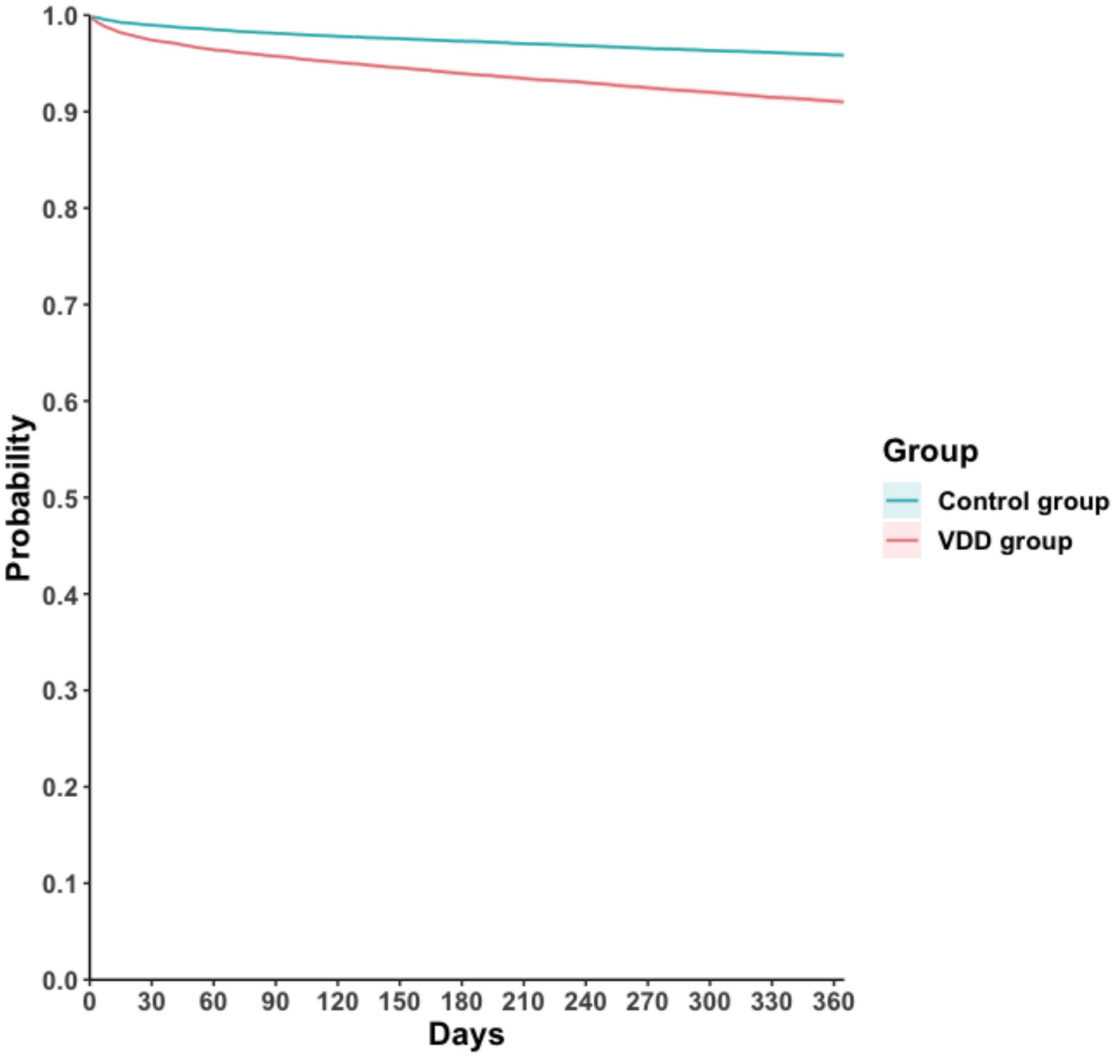
Kaplan–Meier time-to-event free curves of the major adverse kidney event. VDD, vitamin D deficiency.

**Figure 3:**
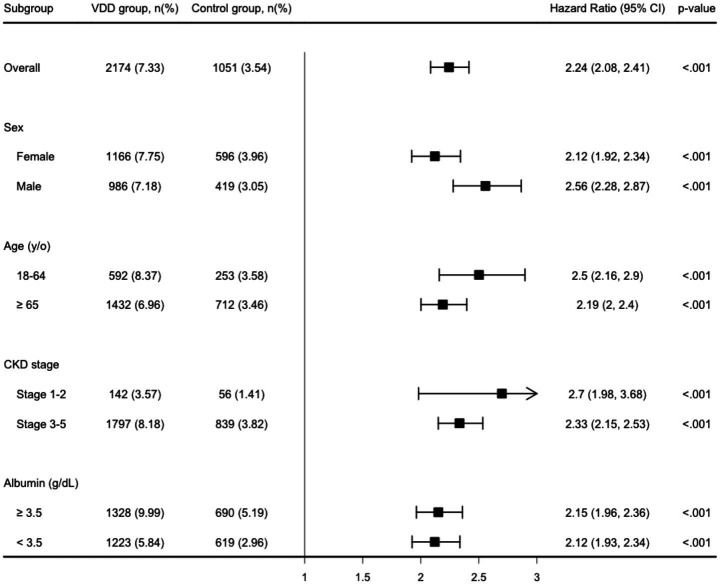
Stratified analysis of the major adverse kidney event. y/o, years old; VDD, vitamin D deficiency.

During the one-year follow-up period, the cumulative incidence of MAKEs was higher in the VDD group compared to the control group (7.3 vs. 3.6 events per 100 person-years), resulting in a HR of 2.24 (95% CI, 2.08–2.41; *p* < 0.001) (Table 2). Similarly, the Kaplan–Meier curves indicated that the VDD group was associated with significantly higher probability of MAKEs compared to the control group (Log-rank test, *p* < 0.001; Figure 2). The E-value for MAKEs was 3.91 (95% lower confidence limit [LCL], 3.58). In the stratified analysis, the higher incidence of MAKEs in the VDD group persisted across all examined subgroups (*p* < 0.001) (Figure 3). Among participants aged 18–64 years, the HR was 2.50 (95% CI, 2.16–2.90), and for those ≥65 years, the HR was 2.19 (95% CI, 2.00–2.40). A similar trend was observed when stratified by sex: males had an HR of 2.12 (95% CI, 1.92–2.34), whereas females had a slightly higher HR of 2.56 (95% CI, 2.28–2.87). Participants with early-stage CKD (stages 1–2) also demonstrated an elevated HR of 2.70 (95% CI, 1.98–3.68), which was consistent among those with more advanced CKD (stages 3–5; HR, 2.33 [95% CI, 2.15–2.53]). Finally, the association remained similar regardless of albumin level (>3.5 g/dL vs. ≤3.5 g/dL), with HRs of 2.15 (95% CI, 1.96–2.36) and 2.12 (95% CI, 1.93–2.34), respectively.

**Table 2:** Primary and secondary outcomes between the vitamin D deficiency group and the control group.

Outcome	VDD group (*n* = 29,654)		Control group (*n* = 29,654)		HR (95% CI)	*p* value	E-value (95% LCL)
	Events (*n*)	Incidence rate per 100 person-years	Events (*n*)	Incidence rate per 100 person-years			
Primary outcome							
MAKEs	2,174	7.3	1,051	3.6	2.24 (2.08,2.41)	<0.001	3.91 (3.58)
Secondary outcomes							
All-cause mortality	4,033	13.6	2,262	7.6	1.92 (1.82,2.02)	<0.001	3.52 (3.04)
All-cause hospitalization	3,742	12.6	3,374	11.4	1.19 (1.14,1.25)	<0.001	1.67 (1.54)

MAKE, major adverse kidney event; VDD, vitamin D deficiency.

### Secondary outcomes

All-cause mortality was observed at an incidence rate of 13.6 events per 100 person-years in the VDD group, compared to 7.6 per 100 person-years in the control group, corresponding to an HR of 1.92 (95% CI, 1.82–2.02; *p* < 0.001) and an E-value of 3.52 (95% LCL, 3.04) (Table 2). In addition, all-cause hospitalization occurred more frequently in the VDD group (12.6 vs. 11.4 per 100 person-years), and an HR of 1.19 (95% CI, 1.14–1.25; *p* < 0.001) (Table 2). The E-value for all-cause hospitalization was 1.67 (95% LCL, 1.54).

## Discussion

In this study, we found a significant association between VDD and an increased risk of MAKEs in patients with CKD. This association remained robust across various subgroups, including age, sex, and CKD severity, suggesting a consistent impact of VDD on renal outcomes. Additionally, VDD was linked to increased risks of all-cause mortality and hospitalization. These findings underscore the clinical importance of maintaining adequate vitamin D levels in CKD management, highlighting VDD as a potentially modifiable risk factor for adverse renal outcomes.

Our results align with previous reviews, demonstrating that VDD contributes to CKD progression and adverse outcomes (5, 31). A notable strength of our study is that we used MAKEs as the primary endpoint, offering a robust and clinically relevant composite metric (i.e., ESKD, urgent dialysis, or dialysis dependence) for evaluating the cumulative burden of advanced renal events. By capturing both progression to ESKD and the need for dialysis in one measure, MAKEs provide a comprehensive hard outcome that underscores the real-world impact of VDD on kidney disease severity.

Nonetheless, minimal associations between VDD and adverse renal outcomes have been reported in certain cohorts. For example, Lunyera et al. (32), focusing on Black Americans from the Jackson Heart Study, found that vitamin D–binding protein (DBP) genotypes and other racial or genetic modifiers might confound the link between 25(OH)D and CKD progression. That study highlights how race-specific or genotype-specific factors can obscure or weaken the observed relationship between VDD and kidney function decline. Our current analysis, by contrast, drew on a large, mixed population from multiple centers, supporting a more consistent association between VDD and worse CKD outcomes overall. Given the variability noted in genetically diverse cohorts, additional research on DBP genetic variants and ancestry-specific differences in vitamin D metabolism is warranted to further clarify the nuanced interplay between VDD and kidney disease risk.

Several biological mechanisms could explain why VDD in CKD is associated with higher MAKEs. Vitamin D is biologically active in multiple organ systems, and its absence may exacerbate pathophysiological processes in CKD. One important mechanism involves the RAAS. Active vitamin D normally suppresses renin expression; when vitamin D is deficient, this suppression is removed, leading to heightened RAAS activity, hypertension, and glomerular hyperfiltration injury (33, 34). Chronic RAAS overactivity contributes to progressive nephron damage and fibrosis, promoting CKD progression to ESKD. As a result, VDD could accelerate MAKEs by worsening blood pressure control and intraglomerular pressure, hastening renal function decline (35).

Another mechanism centers on pro-inflammatory and immune dysregulation. Vitamin D has immunomodulatory effects, partly by inhibiting the nuclear factor kappa B (NF-κB) pathway and reducing inflammatory cytokine release (36, 37). In deficiency states, there may be increased inflammation within the kidneys, as immune cells in VDD patients can adopt a more pro-inflammatory profile and potentially cause ongoing renal injury. Elevated inflammation and oxidative stress can scar renal tissues, thus linking low vitamin D to an increased risk of MAKEs via inflammatory kidney damage (38).

Podocyte injury and proteinuria also represent key pathways. The vitamin D receptor is expressed in podocytes, specialized cells in the kidney’s filtering units, and vitamin D signaling helps maintain podocyte health and the glomerular filtration barrier (39). VDD is associated with a higher prevalence of albuminuria (39). Loss of vitamin D’s protective effects on podocytes can lead to worsening proteinuria, which itself is a major risk factor for CKD progression and adverse outcomes. Experimental studies have shown that active vitamin D analogs, such as derivatives of calcitriol, can reduce proteinuria and prevent podocyte apoptosis in kidney disease models (40). Therefore, in deficient patients, the lack of these renoprotective effects can contribute to a faster GFR decline and more MAKEs.

A further mechanism involves mineral and bone disorder (MBD) and vascular damage. In CKD, VDD contributes to secondary hyperparathyroidism and elevated fibroblast growth factor 23 (FGF23) levels as the body attempts to maintain mineral homeostasis (41, 42). Chronic elevation of parathyroid hormone and FGF23 can induce vascular calcification and cardiac hypertrophy, compounding cardiovascular risk in CKD (43, 44). These changes may indirectly affect kidney health as well, for instance, vascular calcifications can impair renal perfusion. Additionally, skeletal resistance and bone turnover abnormalities can release factors that negatively impact the kidneys. In this way, VDD aggravates CKD-mineral bone disorder, hastening vascular and renal deterioration and increasing the likelihood of MAKEs. These proposed mechanisms are consistent with current clinical consensus. Guidelines have highlighted the multifactorial role of vitamin D in CKD, particularly its influence on mineral metabolism, parathyroid hormone regulation, inflammation, and cardiovascular risk. Disruptions in the vitamin D axis, such as reduced 1α-hydroxylase activity, increased fibroblast growth factor 23, and early onset of secondary hyperparathyroidism, are recognized contributors to renal disease progression and adverse outcomes in CKD patients with VDD (45, 46). Our findings carry important clinical implications. VDD represents a potentially modifiable risk factor in CKD. Unlike fixed risk factors such as age or genetic predispositions, vitamin D status can be improved through supplementation or lifestyle changes. If a causal relationship is confirmed, treating VDD in CKD patients could become a straightforward strategy to reduce the risk of major adverse renal outcomes. In practice, this means clinicians should remain vigilant in screening for VDD in CKD populations and consider repletion therapy when levels are low. For example, using cholecalciferol or ergocalciferol for VDD, or active vitamin D analogs if needed for severe secondary hyperparathyroidism. Current clinical guidelines acknowledge this need, the 2017 KDIGO CKD-MBD update suggests that in CKD stages G3a–G5, VDD or vitamin D insufficiency should be corrected using the same strategies as for the general population (41). Traditionally, the motivation for vitamin D supplementation in CKD has been bone health and controlling secondary hyperparathyroidism. Our study suggests that maintaining sufficient vitamin D might also confer nephroprotective benefits, potentially lowering MAKEs risk. This strengthens the rationale for ensuring vitamin D adequacy as part of comprehensive CKD care.

This study had several strengths. First, this is a large retrospective cohort, which could enhance statistical power and generalizability, and also provide more stratified analyses and robustness findings. Second, PSM was well conducted to minimize the impact of measured confounders. Third, for those unmeasured confounders, E-values indicated that only a small effect could be affected by unmeasured confounders. Last, real-world data from EMRs were used, which reflected the true complexity and heterogeneity of patients, making the results more applicable in real-world settings. Furthermore, the selection of MAKEs as the primary composite endpoint adds clinical relevance, as it directly reflects the most critical outcomes for CKD patients. In addition, our discussion incorporated multiple plausible biological mechanisms, which strengthens the interpretability of the observed associations.

This study also had some limitations. First, the observational design precluded definitive causal inferences. Second, although propensity score matching was used to balance numerous baseline differences between the groups, certain factors, such as dietary intake, supplement use, sun exposure duration, and the timing of vitamin D measurement, could not be accounted for in our analysis. Third, reliance on EMRs and administrative billing codes may have introduced misclassification bias in both CKD diagnosis and vitamin D measurements. Nonetheless, we expect these inaccuracies were comparable across groups, resulting in estimates that tend toward the null value (47). Fourth, although focusing on one-year outcomes is clinically relevant, it may not fully capture the long-term impact of VDD on disease progression and survival. An extended follow-up period would likely yield additional insights into risk magnitude and other clinically significant endpoints. Finally, patients with vitamin D insufficiency (21–29 ng/mL) were excluded to create a clear contrast between deficiency and sufficiency. However, because this group is highly prevalent in clinical practice, our findings may have limited applicability to such patients.

## Conclusion

The study strengthens the evidence that VDD is not merely a biochemical abnormality in CKD, but a condition with tangible impacts on patient outcomes such as MAKEs. Addressing VDD in CKD care, through vigilant monitoring and appropriate repletion, could be a step toward improving the prognosis of patients with chronic kidney disease. Future studies will be crucial to determine whether this association is causal and if vitamin D–targeted interventions can favorably alter the course of CKD.

## Data Availability

The original contributions presented in the study are included in the article/Supplementary material, further inquiries can be directed to the corresponding author.
