# Supplementary material for: Major adverse kidney events among chronic kidney disease patients with vitamin D deficiency
Source: Front Nutr. 2025 Oct 7;12:1650514. doi: 10.3389/fnut.2025.1650514 (PMC12537428; doi:10.3389/fnut.2025.1650514)
Supplement: Supplementary file 1 [file Table_1.DOCX]

**Table S1.** Demographic, diagnostic, procedural, medication, visit, and laboratory codes utilized in the definition of the cohorts.

| **Category** | **Code** | **Description** |
| --- | --- | --- |
| **#1 Age at least 18 years old** | | |
| Demographics | Age | Age (at least 18 years) |
| **#2 Visited HCOs at least twice between Jan 01, 2010 and Jun, 31 2025** | | |
| Visit | Visit | Visit |
| **#3 Diagnosed with CKD** | | |
| Diagnosis | UMLS:ICD10CM:N18 | Chronic kidney disease |
| **#4 Had a vitamin D test within 3 months before the diagnosis of CKD (index date)** | | |
| Labs | LONIC:9034 | Calcidiol (Mass/volume) in Serum or Plasma |
| **#5 No MAKEs events occurred before the index date** | | |
| Diagnosis | UMLS:ICD10CM:Z99.2 | Dependence on renal dialysis |
| Diagnosis | UMLS:ICD9CM:39.95 | Hemodialysis |
| Procedure | UMLS:CPT:90945 | Dialysis procedure other than hemodialysis (eg, peritoneal dialysis, hemofiltration, or other continuous renal replacement therapies), with single evaluation by a physician or other qualified health care professional |
| Procedure | UMLS:CPT:1012740 | Dialysis Services and Procedures |
| Procedure | UMLS:CPT:1006747 | Hemodialysis Access, Intervascular Cannulation for Extracorporeal Circulation, or Shunt Insertion Procedures on Arteries and Veins |
| Procedure | UMLS:CPT:1012752 | Hemodialysis Procedures |
| Diagnosis | UMLS:ICD10CM:N18.6 | End stage renal disease |
| Labs | LONIC:8001 | Estimated glomerular filtration rate by Creatinine-based formula (MDRD) <5 |
| **#6 Have a follow-up record after the index date** | | |
| Visit | Visit | Visit |
| Deceased | Deceased | Deceased |
| Diagnosis | UMLS:ICD10CM:R99 | lll-defined and unknown cause of mortality |

CPT, Current Procedural Terminology; ICD9CM, International Classification of Diseases, ninth Revision, Clinical Modification; ICD10CM, International Classification of Diseases, Tenth Revision, Clinical Modification; LONIC, Logical Observation Identifiers Names and Codes; NLM, National Library of Medicine; RXNORM, medical prescription normalized; UMLS, Unified Medical Language System

**Table S2.** Definitions of covariates coding used in this study.

| **Code** | **Description** |
| --- | --- |
| Age at Index | Age at Index |
| Male | Male |
| Female | Female |
| White | White |
| Black or African American | Black or African American |
| Unknown Race | Unknown Race |
| Asian | Asian |
| Other Race | Other Race |
| 9037 | Hemoglobin A1c/Hemoglobin.total in Blood |
| 8001 | Glomerular filtration rate/1.73 sq M.predicted [Volume Rate/Area] in Serum, Plasma or Blood by Creatinine-based formula (MDRD) |
| 9045 | Albumin [Mass/volume] in Serum, Plasma or Blood |
| UMLS:ICD10CM:F17 | Nicotine dependence |
| UMLS:ICD10CM:F10 | Alcohol related disorders |
| UMLS:ICD10CM:E40-E46 | Malnutrition |
| UMLS:ICD10CM:E66 | Overweight and obesity |
| UMLS:ICD10CM:E11 | Type 2 diabetes mellitus |
| UMLS:ICD10CM:I10 | Essential (primary) hypertension |
| UMLS:ICD10CM:E78 | Disorders of lipoprotein metabolism and other lipidemias |
| UMLS:ICD10CM:I20-I25 | Ischemic heart diseases |
| UMLS:ICD10CM:I48 | Atrial fibrillation and flutter |
| UMLS:ICD10CM:I60-I69 | Cerebrovascular diseases |
| UMLS:ICD10CM:J40-J4A | Chronic lower respiratory diseases |
| UMLS:ICD10CM:K70-K77 | Diseases of liver |
| UMLS:ICD10CM:M32 | Systemic lupus erythematosus |
| UMLS:ICD10CM:C00-D49 | Neoplasms |
| NLM:RXNORM:CV100 | Beta blockers |
| NLM:RXNORM:CV700 | Diuretics |
| NLM:RXNORM:CV800 | Ace inhibitors |
| NLM:RXNORM:CV805 | Angiotensin ii inhibitor |
| NLM:RXNORM:A10BK | Sodium-glucose co-transporter 2 inhibitors |
| NLM:RXNORM:A10BJ | Glucagon-like peptide-1 analogues |
| NLM:RXNORM:CV200 | Calcium channel blockers |
| NLM:RXNORM:C10AA | Hmg coa reductase inhibitors |
| NLM:RXNORM:105694 | Epoetin alfa |
| NLM:RXNORM:2562811 | Finerenone |

ICD10CM, International Classification of Diseases, Tenth Revision, Clinical Modification

NLM, National Library of Medicine

RXNORM, medical prescription normalized

UMLS, Unified Medical Language System

**Table S3.** Definitions of outcomes coding used in this study.

| **Code** | **Description** |
| --- | --- |
| **All-cause mortality** | |
| Deceased | Deceased |
| UMLS:ICD10CM:R99 | Ill-defined and unknown cause of mortality |
| **All-cause hospitalization** | |
| Visit | Visit: Observation Encounter |
| Visit | Visit: Inpatient Non-acute |
| Visit | Visit: Inpatient Acute |
| Visit | Visit: Short Stay |
| **Major adverse kidney events** | |
| UMLS:ICD10CM:Z99.2 | Dependence on renal dialysis |
| UMLS:ICD9CM:39.95 | Hemodialysis |
| UMLS:CPT:90945 | Dialysis procedure other than hemodialysis (eg, peritoneal dialysis, hemofiltration, or other continuous renal replacement therapies), with single evaluation by a physician or other qualified health care professional |
| UMLS:CPT:1012740 | Dialysis Services and Procedures |
| UMLS:CPT:1006747 | Hemodialysis Access, Intervascular Cannulation for Extracorporeal Circulation, or Shunt Insertion Procedures on Arteries and Veins |
| UMLS:CPT:1012752 | Hemodialysis Procedures |
| UMLS:ICD10CM:N18.6 | End stage renal disease |
| LONIC:8001 | Estimated glomerular filtration rate by Creatinine-based formula (MDRD) < 5 |

CPT, Current Procedural Terminology; ICD9CM, International Classification of Diseases, ninth Revision, Clinical Modification; ICD10CM, International Classification of Diseases, Tenth Revision, Clinical Modification; NLM, National Library of Medicine; RXNORM, medical prescription normalized; UMLS, Unified Medical Language System
